# Aβ Mediated Diminution of MTT Reduction—An Artefact of Single Cell Culture?

**DOI:** 10.1371/journal.pone.0003236

**Published:** 2008-09-18

**Authors:** Raik Rönicke, Anja Klemm, Jessica Meinhardt, Ulrich H. Schröder, Marcus Fändrich, Klaus G. Reymann

**Affiliations:** 1 Project Group Neuropharmacology, Leibniz Institute for Neurobiology, Magdeburg, Germany; 2 Research Institute for Applied Neurosciences GmbH, Magdeburg, Germany; 3 Leibniz Institute for Age Research (Fritz Lipmann Institute), Jena, Germany; 4 Max Planck Research Unit for Enzymology of Protein Folding, Halle (Saale), Germany; 5 Martin-Luther University Halle-Wittenberg, Halle, Germany; Swiss Federal Institute of Technology Lausanne, Switzerland

## Abstract

The 3-(4,5-dimethylthiazol-2-yl)-2,5-diphenyl-tetrazoliumbromide (MTT) reduction assay is a frequently used and easily reproducible method to measure beta-amyloid (Aβ) toxicity in different types of single cell culture. To our knowledge, the influence of Aβ on MTT reduction has never been tested in more complex tissue. Initially, we reproduced the disturbed MTT reduction in neuron and astroglia primary cell cultures from rats as well as in the BV2 microglia cell line, utilizing four different Aβ species, namely freshly dissolved Aβ (25-35), fibrillar Aβ (1-40), oligomeric Aβ (1-42) and oligomeric Aβ (1-40). In contrast to the findings in single cell cultures, none of these Aβ species altered MTT reduction in rat organotypic hippocampal slice cultures (OHC). Moreover, application of Aβ to acutely isolated hippocampal slices from adult rats and *in vivo* intracerebroventricular injection of Aβ also did not influence the MTT reduction in the respective tissue. Failure of Aβ penetration into the tissue cannot explain the differences between single cells and the more complex brain tissue. Thus electrophysiological investigations disclosed an impairment of long-term potentiation (LTP) in the CA1 region of hippocampal slices from rat by application of oligomeric Aβ (1-40), but not by freshly dissolved Aβ (25-35) or fibrillar Aβ (1-40). In conclusion, the experiments revealed a glaring discrepancy between single cell cultures and complex brain tissue regarding the effect of different Aβ species on MTT reduction. Particularly, the differential effect of oligomeric versus other Aβ forms on LTP was not reflected in the MTT reduction assay. This may indicate that the Aβ oligomer effect on synaptic function reflected by LTP impairment precedes changes in formazane formation rate or that cells embedded in a more natural environment in the tissue are less susceptible to damage by Aβ, raising cautions against the consideration of single cell MTT reduction activity as a reliable assay in Alzheimer's drug discovery studies.

## Introduction

Deposits of beta-amyloid (Aβ) and neurofibrillary tangles are the two pathological hallmarks of Alzheimer's disease. There is recent evidence that soluble Aβ aggregates can impair function, morphology and subsequently the viability of neuronal cells [1]. Based on NADH dependent reduction activity, cells are able to reduce the tetrazolium salt MTT [3-(4,5-dimethylthiazol-2-yl)-2,5-diphenyltetrazolium bromide] into a formazane [2]. Thus, it is widely accepted that the amount of formazane production correlates with both the number and the viability of the cells. The MTT assay is well established for investigations of cellular viability in single cell cultures [3] and tissue slices [4], [5]. The MTT assay is frequently used to evidence Aβ related changes in membrane properties and disturbed cellular viability [6], [7]. The question how Aβ inhibits cellular MTT reduction is still a matter of debate. Based on their findings that Aβ potently inhibits cellular reduction of MTT in cultured rat hippocampal neurons and HeLa cell lines, Kaneko et al. (1995) have hypothesized that Aβ specifically suppresses mitochondrial succinate dehydrogenase [8]. Studies on rat brain tumor cells [9] and astrocytes [10], on the other hand, indicated that Aβ decreases cellular MTT reduction by accelerating the exocytosis of MTT formazan.

Although many *in vitro* findings on Aβ toxicity and competing, protective agents are based on the MTT assay [11]–[14], the influence of Aβ on MTT reduction has never been tested in more complex models than single cell cultures. Organotypic hippocampal slices (OHC) are an *in vitro* model that retains the three dimensional structure of *in vivo* systems and ranges in complexity between primary cell cultures and intact animals [15]. OHCs represent a well established tool for the investigation of brain damage due to oxygen glucose deprivation (OGD) [16] or epilepsy [17]. When OHCs were exposed to very high doses of Aβ (≥10 µM) neuronal apoptotic cell death [18], [19] and a pronounced activation of astrocytes [20] occurred. More subtle submicromolar Aβ concentrations caused a retraction of neuronal dendrites and a degeneration of dendritic spines [21]. Although it has been shown that MTT is appropriate to evaluate the viability of brain tissue slices and its reduction is impaired after detrimental treatment, such as OGD [5], [22], the influence of Aβ on MTT reduction in OHCs has never been tested before.

In this study, we compared OHCs and primary cell cultures for the effect of different Aβ species, varying in molecule length and aggregation status, on MTT reduction. We used freshly dissolved Aβ (25-35), which is frequently used and already shown to exert detrimental effects on brain function and MTT reduction of single cells well before aggregation occurs [23], [24]. However, we can not exclude aggregation of Aβ (25-35) occurring during the experiment. Further, we used Aβ (1-40) fibrils, which are polypeptide aggregates, characterized by a fibrillar structure and the presence of a cross-β conformation [25]. These fibrils were shown to impair cellular MTT reduction [7]. The third species tested were so-called ‘oligomers’ of Aβ (1-42) [26] and Aβ (1-40) [27]. These oligomers are small non-fibrillar aggregates that are defined by an almost spherical shape and that have been discussed to be early mediators of cellular malfunction within the Alzheimer afflicted brain [28]. Moreover, we analyzed the influence of freshly dissolved Aβ (25-35), fibrillar Aβ (1-40) and oligomeric Aβ (1-40) on long-term potentiation (LTP) the cellular correlate for learning and memory [29] in acute hippocampal slices from rats and compared it with the influence on MTT activity. Surprisingly, in all tissue cultures we could not confirm the Aβ effects on MTT reduction known from primary cell cultures.

## Results

### Aβ impaired MTT reduction in neuronal, astroglia and microglia single cell cultures

**Figure 1:**
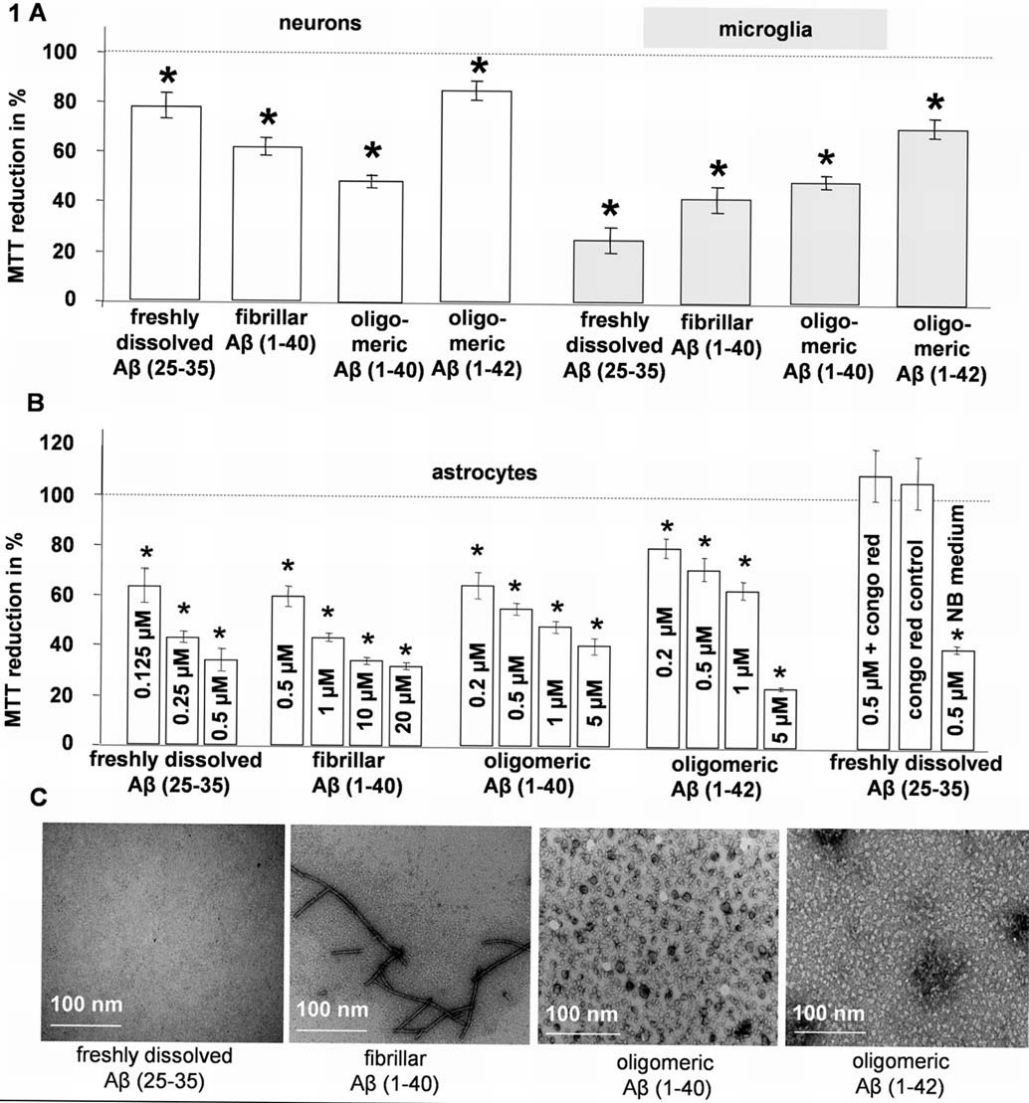
Influence of Aβ on MTT reduction of single cell cultures. A) Influence of Aβ on MTT reduction of neuron and microglia single cell cultures. When applied to cell cultures for 3 days, at 1 µM all Aβ species diminished the MTT reduction significantly in both cell types. The dashed line indicates the control level; * = p≤0.05, Mann–Whitney U-test, n = 10 per group B) Concentration dependent influence of Aβ on MTT reduction activity of astroglia single cell culture. When applied to cell culture for 3 days, any Aβ species diminished the MTT reduction significantly, compared to control. Congo red (2 µM) completely reverses the Aβ effect; Aβ (25-35) diminished the MTT reduction in NB medium, normally used for cultivation of OHC; the dashed line indicates the control level; * = p≤0.05, Mann–Whitney U-test, n = 10 per group C) Electron microscopic images (EMI) revealed that freshly dissolved Aβ (25-35) did not form aggregates. Moreover, EMI conformed the needle like structure of fibrillar Aβ (1-40) and the smaller, spherical shape of oligomeric Aβ (1-40) and oligomeric Aβ (1-42).

We extensively investigated different Aβ species, namely freshly dissolved Aβ (25-35), fibrillar Aβ (1-40), oligomeric Aβ (1-40) and oligomeric Aβ (1-42) for their effects on MTT reduction in neuronal, astroglia and microglia single cell cultures, representing the majority of cell types within the brain. In accordance with the literature [2], [10], each Aβ species led to a pronounced diminution of MTT reduction in all cell types tested (Neurons: control 100±4.4%, Aβ (25-35) 84.4±3.9%, fibrillar Aβ (1-40) 61.1±3.5%, oligomeric Aβ (1-40): 46.0±2.4%, Aβ (1-42) 77.7±5.1%; Microglia: control: 100±5.6%, Aβ (25-35) 25.9±6.2%, fibrillar Aβ (1-40) 42.3±6.5%, oligomeric Aβ (1-40): 49.1±2.5%, Aβ (1-42) 72.7±3.1% Figure 1A). As we intended to investigate the effect of Aβ on MTT reduction in OHCs, where the most abundant cell type is astroglia, we determined the Aβ effect in detail in astroglia single cell cultures. Because OHCs and astroglial cultures are cultivated in different culture media we elucidated whether or not the Aβ mediated disruption of MTT reduction is influenced by the culture medium.

All Aβ species tested acted in a dose dependent manner and Aβ (25-35) showed the highest activity (Figure 1B). In agreement with the literature [30], the Aβ effect could be blocked by congo red (Figure 1B). In neurobasal (NB) medium (used for OHC cultivation) the Aβ effect on MTT activity was similar to the results obtained with DMEM (used for single cell culture; 500 nM Aβ (25-35) in DMEM: 43.7±3.7%, 500 nM Aβ (25-35) in NB: 39.4±2.2%; values were normalized to control; Figure 1B).

### Aβ (25-35), Aβ (1-40) and Aβ (1-42) failed to impair MTT reduction in OHC

**Figure 2:**
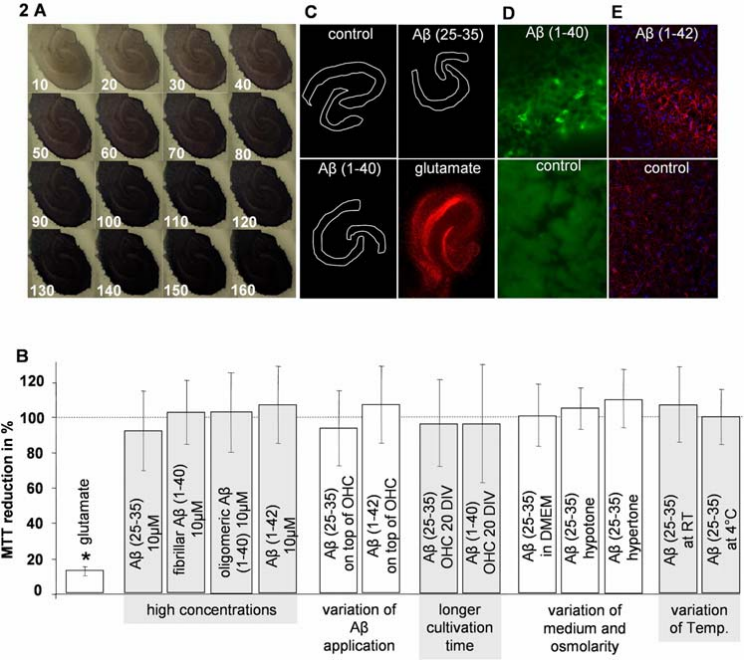
Influence of Aβ on MTT reduction, PI uptake and GFAP expression of OHC. A) Time dependent MTT reduction activity of OHC. Numbers indicate the time after MTT application in minutes B) Influence of glutamate, freshly dissolved Aβ (25-35), fibrillar Aβ (1-40) and oligomeric Aβ (1-42) on MTT reduction activity of OHC under different conditions. Application of 10 µM Aβ for 3–6 days did not diminish the MTT reduction of OHC under different conditions; application of glutamate (15 µM) significantly reduced the MTT reduction, compared to control; the dashed line indicates the control level; * = p≤0.05, Mann–Whitney U-test, n≥12 per group C) PI staining of Aβ and glutamate treated OHCs. Application of 10 µM freshly dissolved Aβ (25-35) and 10 µM fibrillar Aβ (1-40) into the medium for 3 days did not cause cell death. Application of glutamate (15 µM) induced cell death D) Immunostaining of cross sections against fibrillar Aβ (1-40) revealed the presence of Aβ in the slice E) GFAP and DAPI staining of oligomeric Aβ (1-42) treated and control slice. Aβ (1-42) caused an activation of astroglia within the OHC, indicated by an increased GFAP expression.

Compared to single cells, the MTT reduction in OHCs was less frequently investigated. Therefore, we characterized the MTT assay in our system and examined its practicability to measure cell toxicity in OHCs. Similar to single cells, OHC produced the first formazane crystals immediately after MTT application and the reaction was saturated within 3 hours (Figure 2A). The MTT activity was diminished to 17.5±3.4% by application of 15 mM glutamate (Figure 2B). Since this is an approved model for excitotoxicity related cell damage [17], we considered the MTT reduction assay to be suitable for the detection of cell damage in OHCs.

As we intended to reproduce the Aβ effect from single cells in OHCs we applied high concentrations of freshly dissolved Aβ (25-35), fibrillar Aβ (1-40), oligomeric Aβ (1-40) and oligomeric Aβ (1-42). Surprisingly, no Aβ species caused an effect on MTT reduction, independent from the cell culture medium (Aβ (25-35) in NB: 93.4±22.1%; in DMEM: 100.9±19.1%; Figure 2B) and the kind of application (in the medium: Aβ (25-35) 93.4±22.1%, fibrillar Aβ (1-40) 103.2%±17.5%, oligomeric Aβ (1-40) 103.4%±22.6% on top of the slice Aβ (25-35): 94.9%±25.3%; Aβ (1-42) 106.5%±19.3%); Figure 2B). Similar results were obtained for slices that were cultivated for a longer time period (20 DIV), ruling out the possibility that older and less viable slices are more susceptible to Aβ (aged OHCs: Aβ (25-35) 96.7±24.1%, Aβ (1-40) 96.6%±34.5%; Figure 2B).

Succinate dehydrogenase activity [31] and exocytotic processes [32] are temperature-dependent and exocytosis is influenced by osmotic forces [33]. In order to exclude that temperature and osmolarity modify the Aβ effect on MTT reduction in OHCs, we scrutinized the effect of these two parameters. However, lowering the ambient temperature to 21°C or 4°C generally caused a decreased MTT reduction activity of the slices (absolute values not shown), but did not elicit an Aβ-induced diminution in MTT reduction. In addition, the MTT reduction under hypotonic (280 mosmol*kg^−1^-causes a cell swelling) and hypertonic (330 mosmol*kg^−1^-causes a cell shrinkage) conditions was also not significantly altered (Aβ (25-35) hypertone medium 112.9%±15.6%, Aβ (25-35) hypotone medium 107.3%±12.4% of control; Figure 2B). Additionally, we confirmed the missing toxic effect of freshly dissolved Aβ (25-35) and fibrillar Aβ (1-40) in OHCs by an unchanged PI staining and by measuring the release of cytosolic enzyme lactate dehydrogenase (LDH) into the culture supernatant. There was no differences in the PI staining (Figure 2C) and the LDH release of Aβ (25-35) and fibrillar Aβ (1-40) treated slices, compared to control (LDH data not shown).

In order to rule out that diffusion problems due to the size of the Aβ aggregates impede toxicity in the OHCs, we immunostained cross sections of OHCs after Aβ (1-40) treatment. Aβ was clearly marked within the slice (Figure 2D). Furthermore and in line with the literature [20], [34], Aβ (1-42) caused an activation of astroglia, as demonstrated by an increased expression of GFAP (Figure 2E). These results indicate that Aβ was able to affect the astroglia within the OHC, although Aβ failed to disturb the MTT reduction of the slice.

### Separation of single cells from OHCs and treatment with Aβ

**Figure 3:**
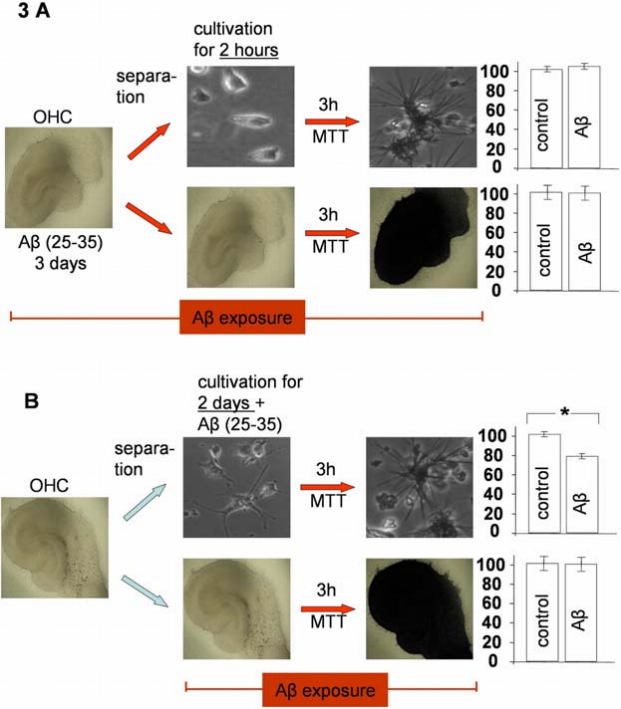
Influence of Aβ on MTT reduction activity of OHC and single cells, generated from OHC. A) Aβ (25-35) 1 µM was applied to the slice for 3 days. The MTT assay was done 2 hours after the preparation of the single cells out of the slice. In this case, 1 µM Aβ did not diminish the MTT reduction of OHC and single cells; B) 1 µM Aβ was applied to the slices and single cells after the preparation for 2 days. In this case, Aβ (25-35) 1 µM significantly reduced the MTT reduction of single cells, compared to control; * = p≤0.05, Mann–Whitney U-test, n = 10 per group.

Considering our conflicting findings in single cells and OHCs it appeared likely that the susceptibility of cells to Aβ mediated diminution of MTT reduction activity depends on their environment. To address this matter, we split one OHC preparation into two groups. One group was cultivated further and the other group was separated into single cells. For the first time we prepared single cells from OHCs. Because of the matured state of the isolated cells only few neurons survived the isolation procedure and thus the cultures consisted largely of astrocytes (Figure 3).

When we exposed the slices to Aβ (25-35) before the separation and measured the MTT reduction activity two hours after the preparation, there was no effect of Aβ (25-35) on the MTT reduction of both, the slices and the single cells (Figure 3A). In contrast, when the slices were first separated and then Aβ (25-35) was applied to the two groups for 2 days, Aβ diminished the MTT reduction in single cell cultures but not in OHCs (Aβ (25-35) 80.1%±1.0% control: 100%±1,1%; Figure 3B).

### Aβ related impairment of LTP is restricted to a particular Aβ species and does not correlate with MTT reduction in acute hippocampal slices

**Figure 4:**
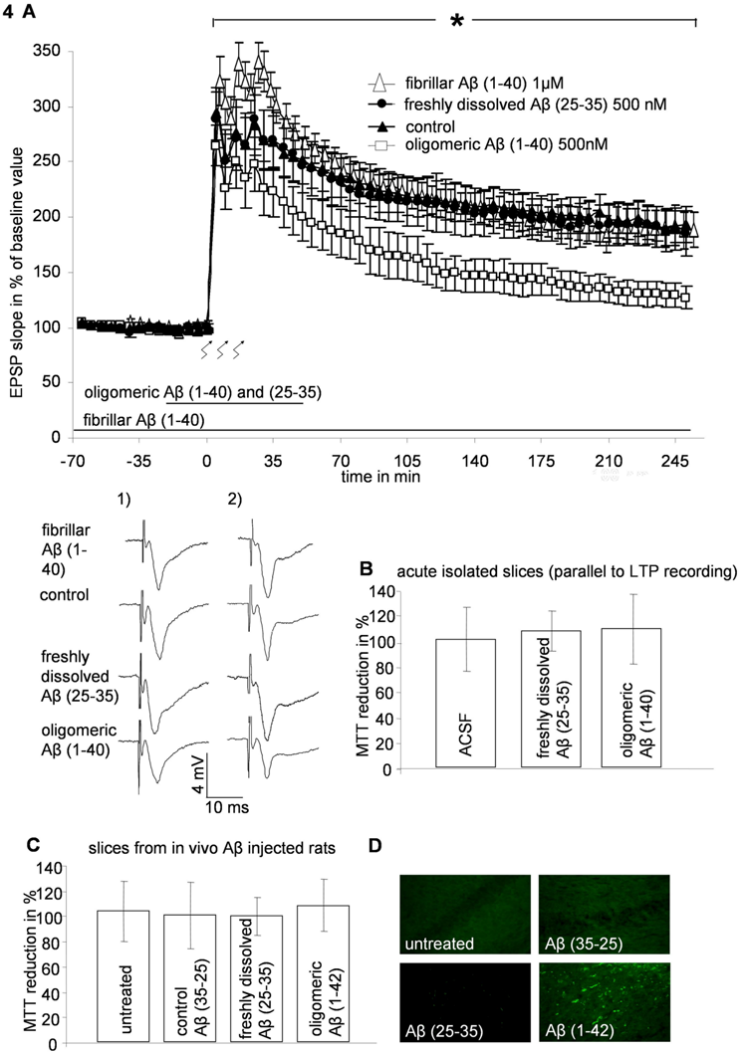
Influence of Aβ on LTP and MTT reduction of acute isolated slices. A) Influence of freshly dissolved Aβ (25-35), oligomeric Aβ (1-40) and fibrillar Aβ (1-40) on LTP of acute hippocampal slices. Oligomeric Aβ (1-40) significantly reduced the LTP, compared to control potentiation. Freshly dissolved Aβ (25-35) and fibrillar Aβ (1-40) did not effect the LTP; * = p≤0.05 ANOVA with repeated measures; The bar indicates the time of Aβ application. Tetanus was applied at time point 0; Analogue traces represent typical recordings of single experiments taken 20 minutes before tetanization (1), and 240 minutes after tetanization (2). B) Aβ treated acute slices did not differ from control slices in their MTT reduction activity. C) Influence of Aβ on MTT reduction activity of *ex vivo* slices. Injection of freshly dissolved Aβ (25-35) and oligomeric Aβ (1-42) for 3 days did not diminish the MTT reduction of the *ex vivo* slices, compared to untreated animals and the reverse control protein Aβ (35-25). D) Immunostaining of cross sections against Aβ revealed the presence of oligomeric Aβ (1-42) in the hippocampus.

To further substantiate the assumption that cells within tissue-like structures react different to Aβ than single cells, we exposed acutely isolated hippocampal slices from adult rats to 500 nM Aβ (25-35) or 500 nM oligomeric Aβ (1-40) or 1 µM fibrillar Aβ (1-40) and measured the influence on LTP. When we exposed slices to Aβ (25-35) and oligomeric Aβ (1-40), 30 min before tetanus application, Aβ (25-35) did not influence the LTP, while application of oligomeric Aβ (1-40) significantly attenuated LTP (oligomeric Aβ (1-40): 139.5%±11.3% n = 8; Aβ (25-35): 184.2%±15.8% n = 8; control: 189.7%±15.9% n = 16 of baseline value 240 min after tetanus application; Figure 4A). Because of their large molecule size Aβ (1-40) fibrils were expected to have limited and slow access to neuronal target structures. Therefore, we exposed slices to Aβ (1-40) fibrils persistently throughout the experiment and with a relatively high concentration of 1 µM. However, application of fibrillar Aβ (1-40) did not alter LTP (fibrillar Aβ (1-40): 187.1±16.6%, n = 8, of baseline value, 240 min after tetanus application; Figure 4A). To investigate whether the disturbed LTP caused by Aβ (1-40) oligomers correlates with a diminished MTT reduction, we applied MTT to acute slices in parallel to LTP recording. There was no difference in the MTT reduction between control, Aβ (25-35) and Aβ (1-40) oligomer treated slices (ACSF control: 100.0±25.0%, Aβ (1-40) 107.9%±27.0%; Aβ (25-35) 106.7%±15.7% Figure 4B).

### Aβ failed to diminish MTT reduction *in vivo*

The short life span of acutely isolated slices from adult animals limits the exposure to Aβ aggregates. OHCs in contrast, allow long-time Aβ exposure but constitute of juvenile tissue. As we could not exclude that longer Aβ applications would indeed be able to reduce cellular viability in mature tissue we injected Aβ (25-35) and oligomeric Aβ (1-42) into the rat brain. Three days after Aβ application, the animals were sacrificed and we measured the MTT reduction in freshly prepared hippocampal slices. Aβ (25-35) and Aβ (1-42) did not influence the MTT reduction in this *in vivo/ex vivo* approach (untreated animal: 103.4±23.7%, Aβ (35-25) control: 100.0%±26.2%, Aβ (1-42) 99.4%±14.9%; Aβ (25-35) 108.0%±20.4%; Figure 4C). To prove whether injected Aβ diffused into the hippocampus, we immunostained cross sections of the *ex vivo* slices. Aβ (1-42) oligomers were clearly marked within the slice (Figure 4D). Hence, an effect of Aβ on hippocampal cells could be expected. However, we could not observe a staining of Aβ (25-35), probably due to a wash out of that protein during the preparation procedure. But when injected Aβ (1-42) oligomers diffuse into the hippocampus, a successful diffusion of the smaller Aβ (25-35) peptide is likely. These data indicate that the missing Aβ effect on MTT reduction in OHC and acute isolated hippocampal slices represent the *in vivo* situation.

## Discussion

In this study we compared the effect of different Aβ species on the MTT reduction activity in hippocampal neurons, astrocytes, microglia, OHCs, acutely isolated hippocampal slices from adult animals and the hippocampal formation *in vivo*. We showed that all tested Aβ species impaired MTT reduction activity in all single cell cultures already at high nanomolar concentrations. These findings are in good agreement with various other studies investigating toxic or activating Aβ effects in hippocampal neurons [35], astrocytes [10] and microglia [36]. In contrast to our findings in the single cell cultures none of the Aβ species affected cellular viability in OHCs, although we could confirm the presence of Aβ in the slices by immunostaining and GFAP upregulation. In line with our observations other studies in OHCs also showed no or, at very high concentrations, only very limited toxic effects of Aβ (25-35), Aβ (1-40) and Aβ (1-42) [18], [19], [37], [38]. In contrast to that and to our findings Lambert et al. published in 1998 that slice cultures could be injured with as little as 5 nM soluble Aβ (1-42) of so called Aβ derived diffusible ligands (ADDL) [39]. Later, Chong et al. described in 2006 neuronal cell death in hippocampal brain slices because of Aβ (1-42) oligomer treatment [40]. The reason for the difference to our results could be the kind of Aβ (1-42) preparation, as both groups used aggregation protocols which resulted in spheres of approximately similar size. However, their contrasting observations render it likely that their mode of preparation resulted in a different internal structure of the aggregates. Future studies should be carried out to extensively compare the different Aβ species for their potentially different effects. Nevertheless, we observed comparable detrimental effects of all investigated Aβ species on MTT reduction in single cell culture, which could not be seen in any complex tissue. That discrepancy between single cells and OHCs regarding the effect of Aβ is difficult to reconcile. As single cell cultures are almost exclusively prepared from embryonic tissue and as OHCs represent juvenile tissue one explanation could be that the respective cells are in different physiological states. Scrutinizing this assumption we show that single cells obtained from juvenile OHCs are only susceptible to Aβ effects after being cultured. Similarly, Yankner et al. (1990) reported that dissociated neurons maintained in cultures are resistant to Aβ (25-35) toxicity during the first days in culture and that Aβ neurotoxicity increases with the age of the culture [41]. This may indicate that cultured cells and cells that are embedded in the intact hippocampal synaptic circuitry and anatomy differ regarding cell properties which are crucial for Aβ toxicity or that the interaction between the neural elements in the relatively intact tissue enables a counteracting protective mechanism. Possible mechanisms may be alterations in the membrane lipid composition [42] or an altered accessibility of lipid rafts for Aβ [43]. Similar reasons may account for the Aβ effects in studies where OHCs were cultured for several weeks [44]. These findings do not reflect the situation in adult tissue as we and others [45] did not observe a fast toxic effect of Aβ after *in vivo* application. Also consistent with our results Geula et al. (1998) did not observe a significant Aβ toxicity in aged rats but found age-dependent Aβ toxicity in aged monkeys [46]. This does not exclude that the hippocampal neurons in OHCs, acutely isolated slices and *in vivo* are physiologically impaired, as LTP was disturbed in the acutely isolated slice preparations at least after Aβ oligomer application. Recent studies increasingly indicated that soluble, pre-fibrillar Aβ assemblies rather than mature fibrils may induce early neuronal alterations, leading to physiological interruption before cell death is detectable [47]. Our LTP experiments elucidated the effects of distinct Aβ species on synaptic potentiation. We show that Aβ (1-40) oligomers disturbed LTP, whereas Aβ (1-40) fibrils did not impair LTP, although Aβ (1-40) fibrils where higher concentrated and permanently exposed to the slices. This is in good agreement with the current view that Aβ oligomers are responsible for the early disturbance of brain physiology [48]–[51]. Whether or not LTP disturbances are a first sign of neuronal degeneration remains to be elucidated. If so, the MTT assay would evidently be unable to detect such early alterations in cellular physiology, as we demonstrated that Aβ (1-40) oligomer mediated LTP disruption was not reflected by MTT reduction in slices. On the other hand, studies utilizing primary neuronal and astroglial cultures showed an inhibition of MTT reduction already 2 h after Aβ application [10], [52]. This may not necessarily reflect cell death, as Aβ-induced alterations in MTT reduction in human cortical cultures could not be confirmed with other cytotoxicity assays like LDH and alamarBlue [53].

Aβ (25-35) did not affect LTP in the present study, although a diminution in LTP was found by others [54]. One possible explanation for this discrepancy is the strain dependence of the Aβ (25-35) effect, as Gengler (2007) showed that the influence of Aβ (25-35) on LTP in rat depends on their genetic background [55].

Taken together, we showed that single cell cultures are prone to impairment by Aβ, whereas cells embedded in the intact hippocampal synaptic circuitry and anatomy are quite resistant, suggesting that results obtained with cell cultures cannot be conferred directly to complex tissue. In addition, we demonstrated that Aβ mediated LTP disruption depends on the Aβ species and does not correlate with MTT reduction in acute isolated slices, relativizing the MTT assay as a reporter of early physiological disruption and drug testing. Thus, Aβ effects observed in single cell cultures should be interpreted cautiously regarding their relevance for more complex brain tissue, independently whether MTT reflects cellular viability or precedes cell death.

## Methods

### Single cell culture

The animals were maintained under constant environmental conditions, with an ambient temperature of 21±2°C, a relative humidity of 40%, a 12-h light–dark cycle and free access to food and water. All animal procedures have been approved by the ethics committee of the German federal state of Sachsen-Anhalt, and are in accordance with the European Communities Council Directive (86/609/EEC).

Cells cultures from 1-day-old Wistar rats (Institute breeding stock) were prepared and cultured as described previously [56]. Briefly, newborn rats were decapitated, and the brains were removed and collected in ice-cold Hanks-buffer solution (Biochrom; Berlin, Germany). The brains were gently passed through nylon meshes of 250 mm and 136 mm pore width, in consecutive order. The cell suspension was centrifuged at 4°C for 5 min at 500g. The cells were resuspended in 10 ml growth medium (DMEM supplemented with 10% (v*v^−1^) fetal calf serum, 20 U*ml^−1^ penicillin and 20 mg*ml^−1^ streptomycin).

Single cells from OHCs were isolated by gently removing the slices from the membrane and collecting them in ice-cold Hanks-buffer solution (Biochrom; Berlin, Germany). Then the protocol for cell culture preparation described above was applied. Preparation and cultivation of OHCs was done as described below.

For astrocyte-enriched cultures (95% astrocytes), cells were seeded in 48 well plates at a starting density of 2*10^4^ cells/ml in DMEM supplemented with 10% (v*v^−1^) fetal calf serum and incubated at 37°C in an atmosphere containing 5% CO_2_. The medium was changed every second day. For neuron-enriched culture (80% neurones), the DMEM was exchanged by Start V Medium (Biochrom) 24 h after seeding.

The cell lineage BV2 microglia was cultured in DMEM supplemented with 10% FBS, 1% Pen/Strep (Biochrom), 1% l-Glutamin (Biochrom) at a density not exceeding 5*10^5^ cells*ml^−1^ and maintained in 5% CO_2_ at 37°C.

### Aβ application/MTT assay

Aβ (25-35) (Bachem) was freshly dissolved in bidistilled water to a concentration of 1 mg*ml^−1^. For fibril formation, recombinant Aβ (1-40) [57] was dissolved in bidistilled water to a concentration of 1 mg*ml^−1^ and incubated for 5–7 days at 37°C. The formation of fibrils was verified by negative stain electron microscopy. Aβ (1-42) oligomers were generated as described [26]. The quality of the oligomer preparation was controlled by negative stain electron microscopy and Sodiumdodecylsulfate-Polyacrylamidgelelectrophoresis (SDS-PAGE). The Aβ species were added to the cell culture medium at a concentration of 0.5–10 µM (Aβ (1-42) oligomers) or 0.5–20 µM (Aβ (1-40) fibrils) and incubated for 1–3 days. Then MTT (Carl Roth) was added to the medium (0.5 mg*ml^−1^) and incubated for 3 hours. The medium was removed and the cells were diluted in 20% SDS/50% Dimethylformamid. The relative formazane concentration was measured by determination of the absorbance at 570 nm (well plate reader, Optima FluoStar).

### Organotypic cultures

Organotypic hippocampal interface slice cultures from 10-day-old Wistar rats (Institute breeding stock) were prepared and cultured as interface slices as described previously [59]. Briefly, the slices were placed on membrane inserts in 6-well plates (NUNC, Wiesbaden, Germany) containing 1.2 ml of NB medium/well and were maintained in a humidified incubator for 12–15 days *in vitro* (DIV).

### Immunhistochemistry

For the immunohistochemical staining of Aβ and GFAP, the slices were fixed in 0.1 M phosphate buffer containing 4% paraformaldehyde. The slices were stored in the fixative overnight. After cryoprotection in 30% sucrose, the slices were rapidly frozen in methylbutane at −80°C. The whole slices were cut on a sliding microtome and the 20 µm sections were stored at 4°C in cryoprotectant (CPS) containing 25% ethylene glycol, 25% glycerine in 0.1 M phosphate-buffered saline (PBS). The slices were transferred from CPS to 0.1 M phosphate buffer and washed overnight. Unspecific bindings were blocked for 2 h in the corresponding serum and then the slices were incubated with the primary antibodies and stored at 4°C overnight. All secondary antibodies were incubated at room temperature for 2 h. The slices were then coverslipped with 1,3-diethyl-8-phenylxanthine (DPX). The following primary antibodies and final dilutions were used: monoclonal mouse anti-GFAP (1∶200; Chemicon), polyclonal chicken anti-Aβ (1∶500; abcam), DAPI (1∶10000; MoBiTec). The primary antibodies were diluted in 0.1 M PBS/0.5% Triton X-100 and 3% donkey normal serum (Sigma, Deisenhofen, Germany). The following secondary antibodies and final dilutions were used: donkey anti-mouse Cy3 (1∶500; Dianova), donkey anti-chicken Cy2 (1∶100; Dianova). These secondary antibodies were diluted in 0.1 M PBS.

### Aβ application/MTT assay/PI staining

The Aβ species were added to the slice culture medium at the respective concentrations (1–10 µM) and incubated for 3–6 days. For the application “on top of the slice”, 1 µl of the Aβ stock solution was directly applicated onto the surface of the slice. 1 µl of the solvent was applicated onto the control slices. Then MTT was applied to the medium (0,5 mg*ml^−1^) and incubated for 3 hours. The slices were quickly removed from the membrane and completely diluted in 20% SDS/50% dimethylformamid (incubation for 24 h at RT). After centrifugation, the relative formazane concentration of the supernatant was measured by determination of the absorbance at 570 nm (well plate reader, Optima FluoStar).

Electron microscopy was done as previously described by [60].

Cell death was evaluated by cellular incorporation of propidium iodide (PI) 3d and 6d after Aβ treatment. Cultures were incubated with PI-containing medium (10 µM) for 2 h at 33°C. Fluorescent images were acquired semiautomatized (Nikon motorized stage; LUCIA software) and analyzed by densitometry to quantify necrotic cell death (LUCIA Image analysis software).

### Acute hippocampal slices/LTP

Hippocampal slices (400 µm thick) were prepared from 7- to 8-week-old male Wistar rats (Institute breeding stock) as described previously [61]. Briefly, both hippocampi were isolated and transferred into a submerged-type recording chamber where they were allowed to recover for at least 1 h before the experiment started. The chamber was constantly perfused with artificial cerebrospinal fluid (ACSF) at a rate of 2.5 ml/min at 33±1°C.

Synaptic responses were elicited by stimulation of the Schaffer collateral–commissural fibers in the stratum radiatum of the CA1 region using lacquer-coated stainless steel stimulating electrodes. Glass electrodes (filled with ACSF, 1–4 MΩ) were placed in the apical dendritic layer to record field excitatory postsynaptic potentials (fEPSPs). The initial slope of the fEPSP was used as a measure of this potential. The stimulus strength of the test pulses was adjusted to 30% of the EPSP maximum. During baseline recording, single stimuli were applied every minute (0,0166 Hz) and were averaged every 5 min. Once a stable baseline had been established, long-term potentiation was induced by applying 100 pulses at an interval of 10 ms and a width of the single pulses of 0.2 ms (strong tetanus) three times at 10 min intervals.

Aβ (1-40) oligomers and fibrils were prepared as described previously [27] and visualized by negative stain electron microscopy. Immediately after the slice preparation, fibrillar Aβ (1-40) was persistently applied to the slices at a concentration of 1 µM. Aβ (1-40) oligomers and Aβ (25-35) were applied to the slice for 30 min before tetanus application at a concentration of 500 nM. The Aβ (1-40) solvent HFIP was removed from the ACSF by exposure to a gentle stream of carbogen for 1h. For control experiments we added the same amount of HFIP used for the Aβ (1-40) experiment to the ACSF and removed it by gasification. There was no difference between the potentiation in the HFIP-deprived ACSF and pure ACSF and, therefore, these experiments were pooled. In parallel to the experiments, some slices of the same preparation were separately exposed to Aβ for 3–4 hours and analyzed with MTT assay as described above.

### In vivo infusion of Aβ

*In vivo* infusion was performed as described previously [62]. Briefly, anaesthesia of 10-week-old male Wistar rats (Institute breeding stock) was induced with halothane in a mixture of nitrous oxide and oxygen (50∶50) and maintained with 2–3% halothane (Sigma, Deisenhofen, Germany) via a rat anaesthetic mask (Stölting). The animals were placed in a Kopf stereotaxic frame. Following a midline incision, a burr hole (1 mm in diameter) was drilled into the skull (coordinates: posterior, 0.9 mm from bregma; lateral, 1.7 mm to satura sagittalis) and a 29-gauge cannula was lowered to 4.5 mm below the skull, according to the rat brain atlas of Paxinos and Watson [63]. Aβ (25-35) (1 mg*ml^−1^) or Aβ (1-42) oligomer (1 mg*ml^−1^) was injected intracerebroventricularly in 3-µl sterile solvent over 5 min. After 5 min the cannula was slowly withdrawn. Aβ (35-25) (1 mg*ml^−1^) was used as inactive peptide control. After three days, acute hippocampal slices were prepared as described above, then directly placed on cell culture membranes and the MTT reduction activity analyzed as described above.

### Statistics

Values of LTP recording are given as mean±S.E.M. Values of MTT reduction are given as mean±S.D. As indicated in Results, the Mann–Whitney U-test or the analysis of variance (ANOVA) with repeated measures was used to compare the field potentials between two groups of differentially treated cells or slices, respectively (i.e., control vs. Aβ treatment), where appropriate.
